# A phase transition for the strength of weak ties

**DOI:** 10.1093/pnasnexus/pgag364

**Published:** 2026-10-22

**Authors:** Esha Chatterjee, Sourav Chatterjee

**Affiliations:** Office of Educational Assessment, Santa Clara University, 500 El Camino Real, Santa Clara, CA 95053, USA; Department of Statistics, Stanford University, 390 Jane Stanford Way, Stanford, CA 94305, USA

**Keywords:** weak ties, social networks, triadic closure, transitivity, phase transition

## Abstract

Granovetter’s “strength of weak ties” argument links local triadic closure to the global importance of weak relationships, but it does not by itself quantify when strong ties can or cannot sustain large-scale connectivity. We study the subgraph formed by strong ties under a uniform, edgewise closure condition. A strong-tie graph is called *p*-transitive when, for every ordered adjacent pair (A,B), at least a fraction *p* of the strong neighbors of *B* other than *A* are also strong neighbors of *A*. Under a fixed maximum-degree bound, we prove a sharp structural transition at p=2/3. If p>2/3, every strong-tie component has size bounded by an explicit function of *p* and the degree bounds, independently of the total network size. If p<2/3, bounded-degree *p*-transitive graphs can have arbitrarily large connected strong-tie components. We also revisit three unweighted real-world networks by thresholding a Jaccard neighborhood-overlap proxy for tie strength and plotting global transitivity against normalized size-biased mean component size. These empirical curves show fragmentation as low-overlap edges are removed, but their statistic and thresholds are not the theorem’s edgewise parameter. The theorem is therefore a conditional structural result, while the data illustrate a related clustering–connectivity tradeoff rather than a direct validation of the constant 2/3.

Significance Statement Granovetter’s “strength of weak ties” argument links local clustering to global connectivity, but the structural threshold governing this relationship has remained unclear. We prove that, in bounded-degree networks, uniform edgewise closure above 2/3 forces every strong-tie component to remain bounded independently of total network size, whereas below 2/3 arbitrarily large connected examples exist. Thus the theorem identifies a sharp mathematical transition between fragmentation and possible large-scale connectivity, clarifying when weak ties are structurally important. It makes no causal claim about how social ties form or evolve.

## Introduction

A person’s close family members and closest friends are natural examples of strong ties; a former classmate, a distant professional contact, or an occasional collaborator may be a weak tie. Strong ties often belong to dense social circles. Weak ties, although individually less intense, may connect circles that otherwise have little contact. This tension is the starting point of Granovetter’s classic theory of the strength of weak ties (1).

Granovetter’s local premise is a form of triadic closure: if an individual *A* has strong ties to both *B* and *C*, then *B* and *C* are likely to be tied as well. In the extreme version in which every such closing tie is strong, the connected components formed by strong ties are cliques. Consequently, any tie that connects distant parts of a network cannot be strong; this is the familiar exact-closure argument discussed, for example, by Easley and Kleinberg (2). The more delicate question is quantitative: how much local closure is enough to force every strong-tie component to remain small?

This question is distinct from asking whether weak ties are useful in a particular empirical setting. The empirical importance of weak ties varies with the outcome, the population, and the meaning of tie strength (3–6). Our aim is narrower and mathematical. We ask when a deterministic local condition on the strong-tie subgraph alone implies a network-size-independent bound on the number of vertices reachable through strong ties.

The answer has the form of a phase transition. In network science, this term describes an abrupt qualitative change in large-scale structure as a parameter crosses a critical value, as in the emergence or disappearance of a giant component in random-graph and percolation models (7, 8). Here the parameter is a uniform local closure level *p*. Theorem 1 shows that the critical value is 2/3: above it, bounded degree forces all strong-tie components to have uniformly bounded size; below it, a fixed bounded degree can coexist with arbitrarily large connected strong-tie components. No claim is made at the critical value itself.

The theorem is conditional. It does not model how the parameter *p* arises, how strong and weak ties are formed, or whether adding weak ties causally changes the closure of strong ties. We therefore separate the theorem from our empirical illustrations. In three unweighted networks we remove edges according to a neighborhood-overlap proxy and observe how a standard global clustering statistic changes together with a normalized component-size statistic. The resulting fragmentation curves illustrate a related clustering–connectivity tradeoff, but global transitivity is not the uniform edgewise quantity in Theorem 1, and the filtering procedure itself favors triangle-rich edges. We consequently do not interpret the empirical curves as tests of the theoretical threshold.

## Theoretical result

Let G=(V,E) be the finite, simple, undirected graph whose edges are the strong ties. For v∈V, write T(v) for its set of strong neighbors and d(v)=|T(v)| for its strong-tie degree.

Definition 1 **(Uniform edgewise transitivity)**

For p∈[0,1], the strong ties are **p*-transitive* if, for every ordered adjacent pair (u,v),

(1) |T(u)∩T(v)|≥p(d(v)−1).

Thus, among the strong neighbors of *v* other than *u*, at least a fraction *p* are also strong neighbors of *u*.

Definition 1 is deliberately stronger than standard aggregate clustering measures. It is a uniform requirement on every ordered edge, rather than an average over vertices or triples. In fact, it implies both local and global clustering lower bounds. If t(v) is the number of edges among the neighbors of *v*, then summing (1) over u∈T(v) gives 2t(v)≥pd(v)(d(v)−1), so every local clustering coefficient with d(v)≥2 is at least *p*. Summing over all ordered edges likewise shows that the usual global transitivity, three times the number of triangles divided by the number of connected triples, is at least *p*. Neither converse holds: a graph can have high average or global clustering while containing a bridge or another edge with almost no neighborhood overlap. This distinction is central when comparing the theorem with empirical network summaries.

For A∈V, let N(A) be the number of vertices other than *A* in the connected component of *A* in *G*.

Theorem 1 Assume that the strong ties are *p*-transitive. Suppose that every vertex has degree at most d≥2 and at least $d^{\prime }\geq 0$. Put

$$d_{1}=\max\{d^{\prime }-1,1\},\;d_{2}=\max\{d^{\prime },1\}.$$

If p>2/3, then, for every A∈V,

(2) $$N(A)\leq \frac{d(d+1)(d+(3p-2)d_{1})}{(3p-2)d_{1}d_{2}}-1.$$

Conversely, let p<2/3, and choose any positive integer *m* satisfying

(3) $$p\leq \frac{2m-2}{3m-2}.$$

For every *n*, there is a finite (3m−1)-regular graph with *p*-transitive strong ties such that N(A)>n for every vertex *A*. Consequently, for every p<2/3 there is a degree bound depending only on *p* under which strong-tie components can be arbitrarily large.

The lower-degree information is not required for the first conclusion: one may always set $d^{\prime }=0$, in which case $d_{1}=d_{2}=1$. A positive lower bound can make (2) much smaller. For example, with p=0.9, maximum degree d=20, and minimum degree $d^{\prime }=10$, the bound is N(A)≤174.33…, so fewer than 175 other vertices are reachable through strong ties. With only $d^{\prime }=0$, the corresponding bound is 12,419.

The explicit expression also has a simpler interpretation away from the threshold.

Corollary 2 If p≥2/3+ε with ε>0 and only a maximum degree bound *d* is used, then

$$N(A)\leq \frac{d(d+1)(d+3\varepsilon )}{3\varepsilon }-1\leq \frac{d(d+1{)}^{2}}{3\varepsilon }-1.$$

Thus the component-size bound is $O(d^{3}/\varepsilon )$ for fixed *d* as the closure level stays ε above 2/3.

The bound in Theorem 1 is not asserted to be optimal. At p=1, for instance, exact closure makes every connected component a clique, giving the sharper bound N(A)≤d. The contribution of the theorem is the sharp universal threshold: exact closure can be weakened to any p>2/3 while retaining a network-size-independent conclusion, whereas below 2/3 such a conclusion fails even at a fixed finite degree.

The maximum-degree assumption is essential to this uniform statement. The theorem applies to every finite graph, including graphs with heterogeneous degrees, after taking *d* to be the largest degree. However, for a sequence of networks with hubs whose maximum degree grows with network size, the upper bound can grow as well and need not rule out macroscopic components. Heavy-tailed degree distributions are common in empirical networks (8, 9); extending the result to degree-dependent, probabilistic, or hub-robust hypotheses is an important open direction.

## Related empirical fragmentation patterns

The theorem concerns a designated strong-tie subgraph and a worst-edge closure condition. The three datasets considered here are unweighted and do not label edges as strong or weak. We therefore use a structural proxy and present the results as descriptive illustrations only.

### Thresholding rule and network statistics

For an edge e={u,v} in the original graph $G_{0}$, let


$$S_{u}=T_{G_{0}}(u)\setminus \{v\},\;S_{v}=T_{G_{0}}(v)\setminus \{u\},$$

and define the fixed neighborhood-overlap score


(4) $$s(e)=\frac{\left|S_{u}\cap S_{v}\right|}{\left|S_{u}\cup S_{v}\right|}.$$

If the denominator is zero, we set s(e)=1. This is a Jaccard-type overlap measure used as a proxy for tie strength in network analysis (4). The score is computed once in the full graph. For each distinct threshold *t*, we then form


$$G_{t}=(V,\{e\in E(G_{0})\;:\;s(e)\geq t\}).$$

Using thresholded graphs, rather than deleting tied edges in an arbitrary order, makes the trajectory invariant to the ordering of equal scores.

For each $G_{t}$, we calculate the standard global transitivity $C(G_{t})$ and


$$\chi (G_{t})=\frac{1}{|V{|}^{2}}\sum_{K\in C(G_{t})}|K{|}^{2},$$

where $C(G_{t})$ is the set of connected components. This clarifies the averaging convention: $\left|V|\chi (G_{t}\right)$ is the mean component size seen by a uniformly sampled vertex, which is size-biased relative to sampling components uniformly. Equivalently, $\chi (G_{t})$ is the probability that two independent uniformly sampled vertices lie in the same component. The normalization places all datasets on the common scale [0,1].

There are two built-in reasons not to identify these curves with Theorem 1. First, $C(G_{t})$ is an aggregate clustering coefficient, whereas *p* is a uniform edgewise minimum condition. Second, thresholding by neighborhood overlap mechanically retains triangle-rich edges and therefore tends to increase clustering. The plots can reveal how connectivity and aggregate closure covary under this particular filtration, but they neither estimate *p* nor validate a universal critical value.

### Datasets and results

The Facebook page–page network has 22,470 vertices and 171,002 supplied edge records; 179 self-loops were removed, leaving 170,823 simple edges. Vertices are verified Facebook pages and edges are mutual likes. The graph is part of the MUSAE collection (10). Figure 1 shows a large initial loss of connectivity when the zero-overlap edge batch is removed, followed by a broader decline as transitivity rises. The normalized mean component size is close to zero by a global transitivity of roughly 0.65. This numerical proximity to 2/3 is descriptive and is not treated as agreement with the theorem.

**Figure 1:**
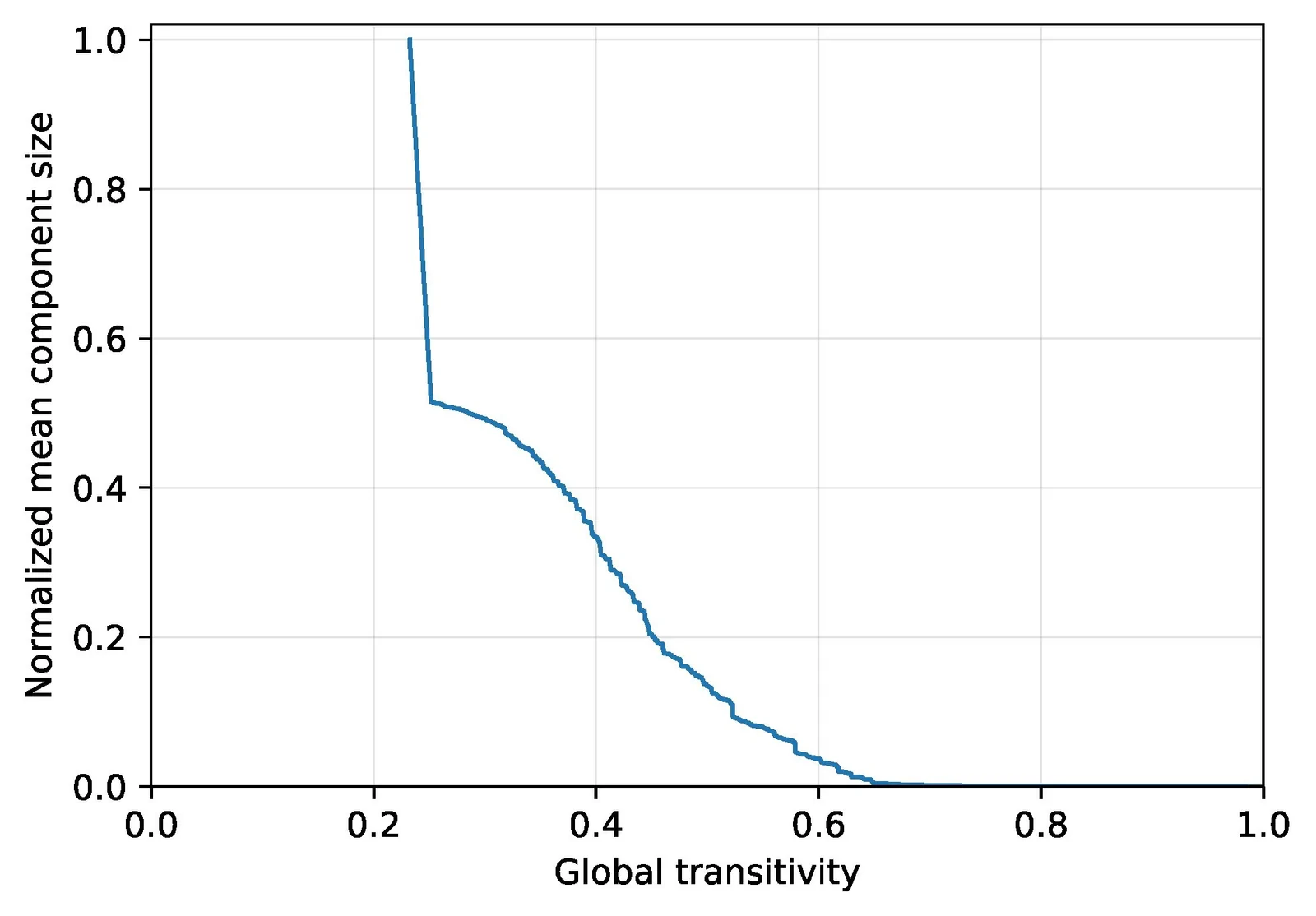
Facebook page–page network. Global transitivity and normalized size-biased mean component size are recorded for the thresholded graphs $G_{t}$ as low-overlap edges are excluded.

The condensed-matter collaboration network contains 16,726 scientists and 47,594 coauthorship edges from preprints posted between January 1995 and December 1999 (11). Edge weights in the source data are ignored here. The full graph is already disconnected, with $C(G_{0})=0.360$ and $\chi (G_{0})=0.687$. Excluding the 2,578 zero-overlap edges raises global transitivity to 0.380 and lowers *χ* to 0.501. The subsequent decline is broad: *χ* falls below 0.01 by global transitivity about 0.77 and below 0.001 near 0.80 (Fig. 2). Collaboration networks are projections of author–paper affiliation data and contain overlapping cliques generated by multi-author papers; this topology can alter both the overlap scores and the shape of the filtration.

**Figure 2:**
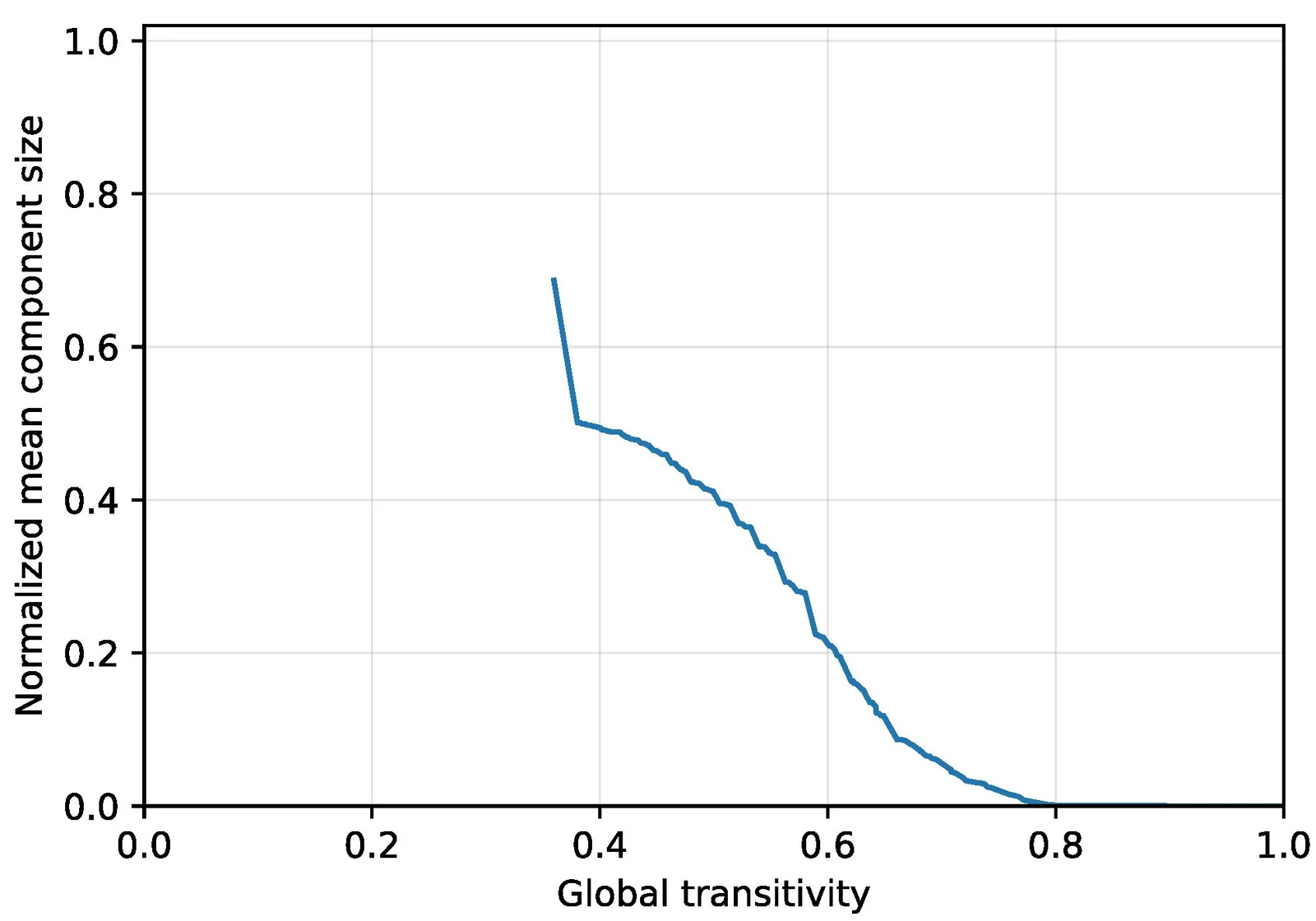
Condensed-matter collaboration network. Removing the zero-overlap edge batch produces the initial drop from χ=0.687 to 0.501; the subsequent decline is broad, with χ<0.01 by global transitivity about 0.77.

The GitHub network has 37,700 developers and 289,003 mutual-follower edges, also from the MUSAE collection (10). As in the Facebook graph, removing the batch of zero-overlap edges produces an immediate drop. The remaining decline is concentrated at substantially lower global-transitivity values than in the collaboration network (Fig. 3).

**Figure 3:**
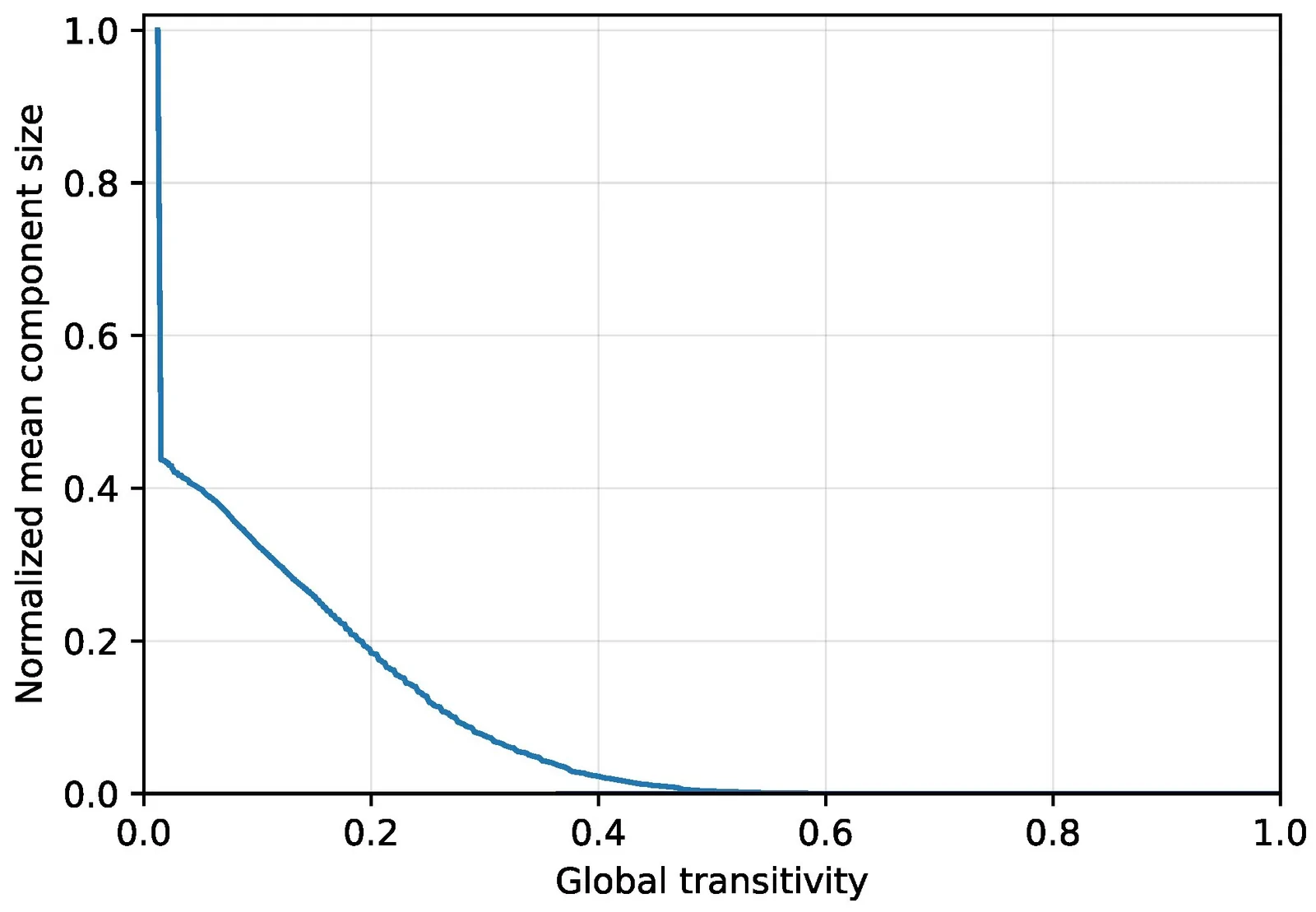
GitHub mutual-follower network. Most of the connectivity loss occurs at global-transitivity values below those in the other two datasets.

Taken together, the three curves support only a qualitative statement: preferentially retaining high-overlap edges trades global reach for triangle-rich local structure. They do not exhibit a common empirical threshold. The location and sharpness of the decline can depend on the degree distribution, community structure, affiliation projection, the mass of zero-overlap edges, and the chosen definition of tie strength. Weighted interaction frequency, communication duration, temporal persistence, embeddedness measures other than Jaccard overlap, or externally measured relationship intensity could all yield different trajectories. Evaluating robustness across such definitions is a separate empirical research program.

## Proof of Theorem 1

We divide the argument into four lemmas: the construction below the threshold, an overlap inequality along a length-two path, a negative-drift estimate above the threshold, and the conversion of that drift into a component-size bound.

Lemma 3 **(Cyclic block construction)**

Let *m* be a positive integer and let N≥4. Arrange *N* disjoint blocks of *m* vertices on a circle (Fig. 4). Join two vertices when their blocks are equal or adjacent on the circle. The resulting graph is (3m−1)-regular and connected. For every edge {u,v},

|T(u)∩T(v)|≥2m−2.

Consequently, it is *p*-transitive for every

$$p\leq \frac{2m-2}{3m-2}.$$

Proof. Every vertex is joined to the other m−1 vertices in its own block and to all 2m vertices in the two adjacent blocks, so its degree is 3m−1. If *u* and *v* are in the same block, their common-neighbor count is larger than the claimed lower bound. If they are in adjacent blocks, their common neighbors are precisely the other vertices in those two blocks, giving 2m−2. Since d(v)−1=3m−2, Definition 1 follows. Connectivity is immediate from the cyclic arrangement.

**Figure 4:**
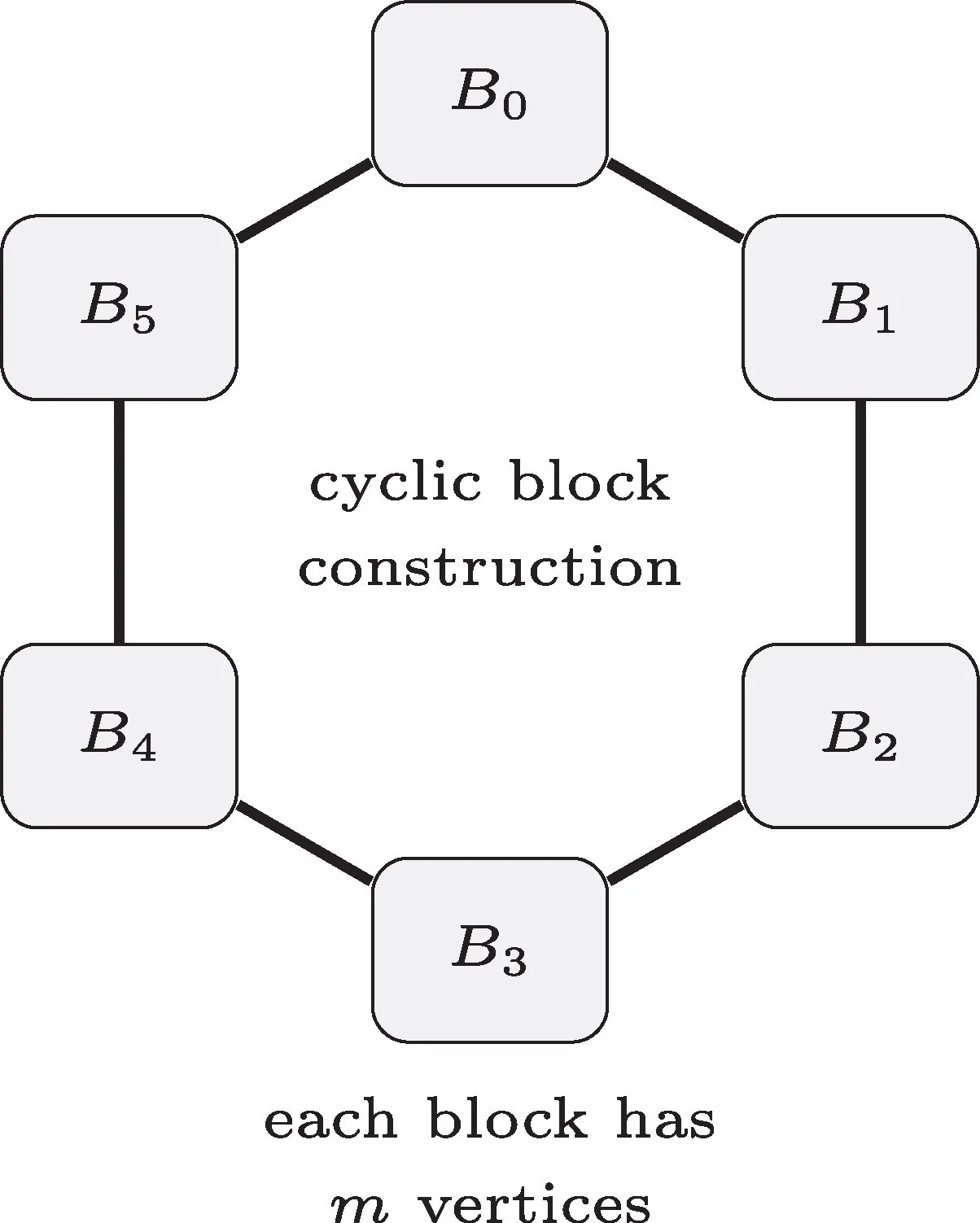
Schematic of the construction in Lemma 3. Each block induces a clique, and every pair of adjacent blocks is joined by all possible edges. The displayed heavy lines represent complete bipartite connections, not single edges.

Lemma 3 proves the converse part of Theorem 1: for any prescribed *n*, choose *N* large enough that Nm−1>n. We now turn to p>2/3.

Lemma 4 **(Overlap across a two-step path)**

Suppose that D,C,B is a strong-tie path. Then

(5) $$\begin{array}{rl} & \left|T(D)\cap T(B)\right|\\ & \;\geq p\max\{d(B)-1,d(C)-1\}+p(d(C)-1)-d(C)+1. \end{array}$$

Proof. Because *C* is adjacent to both *D* and *B* and is not an element of T(C),

$$\begin{array}{rl} \left|T(D)\cap T(B)\right| & \geq |T(D)\cap T(C)\cap T(B)|+1\\ & \geq |T(D)\cap T(C)|+|T(B)\cap T(C)|-|T(C)|+1. \end{array}$$

Applying Definition 1 to the ordered edge (D,C) and to both orientations of the edge *BC* gives

|T(D)∩T(C)|≥p(d(C)−1)

and

|T(B)∩T(C)|≥pmax{d(B)−1,d(C)−1}.

Substitution yields (5).

Fix a root *A* in a strong-tie component, and write δ(x,A) for strong-tie graph distance.

Lemma 5 **(Negative radial drift)**

Let *B* satisfy δ(B,A)≥2, and let $B^{\prime }$ be a uniformly sampled strong neighbor of *B*. Then

(6) $$\begin{array}{rl} & E[\delta (B^{\prime },A)-\delta (B,A)]\\ & \;\leq \frac{\left(2-3p)\max\{d(B)-1,d(C)-1\right\}}{d(B)}, \end{array}$$

where *C* is any neighbor of *B* lying one step closer to *A*. In particular, if p>2/3, d(B)≤d, and all degrees are at least $d^{\prime }$, then

(7) $$E[\delta (B^{\prime },A)-\delta (B,A)]\leq \frac{(2-3p)d_{1}}{d}.$$

Proof. Choose C∈T(B) with δ(C,A)=δ(B,A)−1 and D∈T(C) with δ(D,A)=δ(B,A)−2. A one-step move from *B* changes the distance from *A* by at most one. The distance increases only if $B^{\prime }$ is outside T(C)∪{C}. By *p*-transitivity, the number of such neighbors is at most (1−p)(d(B)−1), and hence

(8) $$P(\delta (B^{\prime },A)-\delta (B,A)=1)\leq \frac{\left(1-p)(d(B)-1\right)}{d(B)}.$$

If $B^{\prime }\in T(D)\cap T(B)$, then the triangle inequality forces $\delta (B^{\prime },A)=\delta (B,A)-1$. Lemma 4 therefore gives a lower bound on the probability of a one-step decrease. Subtracting that bound from (8) yields

$$\begin{array}{rl} E[\delta (B^{\prime },A)-\delta (B,A)] & \leq \frac{1}{d(B)}((1-p)(d(B)-1+d(C)-1)\\ & \;-p\max\{d(B)-1,d(C)-1\})\\ & \leq \frac{\left(2-3p)\max\{d(B)-1,d(C)-1\right\}}{d(B)}, \end{array}$$

which is (6). For (7), note that the maximum in the numerator is at least $d_{1}=\max\{d^{\prime }-1,1\}$, while d(B)≤d. Because 2−3p<0, these inequalities give the stated uniform upper bound.

Lemma 6 **(Stationary mass near the root)**

Under the assumptions of the first part of Theorem 1, let S(A) be the strong-tie component containing *A*. If |S(A)|≥3, then

(9) $$\frac{\sum_{B\in T(A)\cup \{A\}}d(B)}{\sum_{B\in S(A)}d(B)}\geq \frac{(3p-2)d_{1}}{d+(3p-2)d_{1}}.$$

Consequently,

(10) $$|S(A)|\leq \frac{d(d+1)(d+(3p-2)d_{1})}{(3p-2)d_{1}d_{2}}.$$

Proof. Run simple random walk on the connected graph induced by S(A). Every vertex in this component has positive degree, so

$$\pi (B)=\frac{d(B)}{\sum_{C\in S(A)}d(C)},\;B\in S(A),$$

is well defined and is the stationary distribution. Let X∼π, let $X^{\prime }$ be one random-walk step from *X*, and set Δ=δ(X,A) and $\Delta^{\prime }=\delta (X^{\prime },A)$. Stationarity gives $E(\Delta^{\prime }-\Delta )=0$.

When Δ≥2, Lemma 5 gives the upper bound $(2-3p)d_{1}/d$ for the conditional drift. When Δ<2, the drift is at most 1. Writing q=P(Δ<2), we obtain

$$0\leq \frac{(2-3p)d_{1}}{d}(1-q)+q,$$

so

$$q\geq \frac{(3p-2)d_{1}}{d+(3p-2)d_{1}}.$$

Since {B:δ(B,A)<2}=T(A)∪{A}, this is (9).

The numerator on the left side of (9) is at most d(d+1): there are at most d+1 terms and each is at most *d*. Every vertex of S(A) has degree at least $d_{2}=\max\{d^{\prime },1\}$, so the denominator is at least $|S(A)|d_{2}$. Combining these bounds with (9) gives (10).

Proof of Theorem 1. For p<2/3, choose *m* satisfying (3) and apply Lemma 3 with *N* large enough that Nm−1>n.

Now suppose that p>2/3. If N(A)≤1, the conclusion is immediate from the positive right side of (2). Otherwise |S(A)|=N(A)+1≥3, and Lemma 6 applies. Subtracting one from (10) gives (2).

## Conclusion

Theorem 1 gives a precise conditional version of the local-to-global intuition behind the strength of weak ties. Under a fixed degree bound, a uniform strong-tie closure level above 2/3 prevents strong ties alone from supporting components whose size grows with the whole network. Below 2/3, arbitrarily large connected examples exist at a degree bound that depends only on *p*. This sharpens the elementary exact-closure case p=1 and identifies the point at which the universal bounded-component conclusion changes.

The theorem does not explain how closure levels are generated, and it does not establish that a greater prevalence of weak ties raises or lowers the transitivity of strong ties. Such causal statements require a model of tie formation or suitable longitudinal or experimental data. The empirical filtrations reported here make a separate, descriptive point: under a Jaccard-overlap proxy, removing low-overlap edges in three networks produces substantial fragmentation while aggregate clustering rises. The trajectories differ markedly, as one should expect from their different degree distributions, topologies, and the gap between the theorem’s worst-edge hypothesis and a global clustering coefficient.

Natural extensions include degree-sensitive versions that remain informative in the presence of hubs, probabilistic variants that permit a small fraction of exceptional edges, generative models for the joint formation of strong and weak ties, and robustness analyses across alternative empirical measures of tie strength. These directions would connect the sharp structural theorem more directly to variation across observed social environments.

## Data Availability

The public source datasets are identified in the section titled “Datasets and results.” The exact raw Facebook, GitHub, and condensed-matter graph files used here, analysis code, processed trajectories, numerical summaries, and publication-ready figure files will be archived at https://doi.org/10.5281/zenodo.21464149. The repository record will contain the exact software requirements and offline commands needed to reproduce the threshold trajectories and normalized figures from the bundled raw inputs.
